# Alternatives to Cow’s Milk-Based Infant Formulas in the Prevention and Management of Cow’s Milk Allergy

**DOI:** 10.3390/foods11070926

**Published:** 2022-03-23

**Authors:** Natalia Zofia Maryniak, Ana Isabel Sancho, Egon Bech Hansen, Katrine Lindholm Bøgh

**Affiliations:** National Food Institute, Technical University of Denmark, DK-2800 Kgs. Lyngby, Denmark; nazoma@food.dtu.dk (N.Z.M.); anasa@food.dtu.dk (A.I.S.); egbh@food.dtu.dk (E.B.H.)

**Keywords:** infant formula, processing, plant-based proteins, mammalian milk-based proteins, alternative infant formula, cow’s milk allergy, allergy prevention, allergy management

## Abstract

Cow’s milk-based infant formulas are the most common substitute to mother’s milk in infancy when breastfeeding is impossible or insufficient, as cow’s milk is a globally available source of mammalian proteins with high nutritional value. However, cow’s milk allergy (CMA) is the most prevalent type of food allergy among infants, affecting up to 3.8% of small children. Hypoallergenic infant formulas based on hydrolysed cow’s milk proteins are commercially available for the management of CMA. Yet, there is a growing demand for more options for infant feeding, both in general but especially for the prevention and management of CMA. Milk from other mammalian sources than the cow, such as goat, sheep, camel, donkey, and horse, has received some attention in the last decade due to the different protein composition profile and protein amino acid sequences, resulting in a potentially low cross-reactivity with cow’s milk proteins. Recently, proteins from plant sources, such as potato, lentil, chickpeas, quinoa, in addition to soy and rice, have gained increased interest due to their climate friendly and vegan status as well as potential lower allergenicity. In this review, we provide an overview of current and potential future infant formulas and their relevance in CMA prevention and management.

## 1. Introduction

Mother’s milk is constantly changing to adapt to the need of the infant as the infant grows. The composition and nutrients, including proteins, carbohydrates, vitamins, hormones, antibodies, antibacterial agents, growth factors, and cytokines, change according to the infants age for proper development and immune modulation [1]. In contrast, formulas are divided into stage one infant formulas (0–6 months of age), stage two follow-up formulas (6–12 months of age), and stage three toddler formulas (above 12 months of age) to adapt to the need of infants at different stages of development. Breastfeeding, in comparison to use of infant formulas, provides many benefits, such as better brain development and protection against infections as well as obesity [1,2,3]. In general, it is recommended to breastfeed for at least the first 6 months of the infant’s life, further continuing the breastfeeding while introducing complementary foods [4]. Infant formulas are specific products produced as a substitute to mother’s milk for situations where breastfeeding is not possible or is insufficient. They are required to fulfil certain nutritional requirements [5] and are mainly based on cow’s milk proteins. When an infant has been diagnosed with cow’s milk allergy (CMA) and cannot be fully breastfed, the use of a hypoallergenic cow’s milk-based, extensively hydrolysed formula (eHF) is generally recommended for management of the CMA, with amino acid-based infant formula (AAF) as an alternative if the cow’s milk-allergic infants suffer from severe CMA or cannot tolerate the eHF. Infant formulas based on plant proteins are in some countries recommended as a second choice for the management of CMA [1].

At present, in EU, infant formulas can only be based on cow’s and goat milk proteins, soy proteins, as well as hydrolysed proteins [6,7]. Yet, infant formulas based on alternative process-modified versions of cow’s milk proteins, from other mammalian milk, or based on other plant proteins have been suggested and investigated for various reasons. One main interest in providing new and alternative infant formulas is for use in the prevention and management of CMA, as infants suffering from CMA cannot tolerate conventional cow’s milk-based infant formulas and, in some situations, may not even tolerate eHFs [8]. Another reason to search for alternative infant formulas is the increasing interest in plant-based diets connected to environmental, climate, and ethical issues [9,10]. In the present review, we will provide an overview and discuss current and potential future options for infant formulas in the context of CMA prevention and management.

## 2. Food Allergy

Food allergy, which is defined as an immune-mediated adverse reaction to otherwise harmless food proteins [11], is a growing global health problem [12]. More than 70 foods have been reported to induce allergic reactions after their consumption, and eight of them are responsible for more than 90% of all reactions [13,14]. These are peanut, tree nut, cow’s milk, soy, wheat, hen’s egg, fish, and shellfish [14].

Food allergy affects ~1–3% of adults and ~6–8% of small children although the reported prevalence seems to differ between individual studies, countries, and continents [13,15,16]. The prevalence is observed to be higher in small children than in adults because many children naturally outgrow their food allergy over time, gaining tolerance to foods they were previously allergic to [17,18]. There is no unequivocal explanation on why some children outgrow their food allergy while others do not, but several host-, environmental- and allergen-relating factors may be contributing determinants, such as disease severity, gut immune system maturation, gut microbiota composition, type(s) and numbers of culprit allergen(s), or epitope recognition pattern [19,20,21,22].

At present, there are only very limited treatment possibilities, and strict avoidance of the offending foods is the main viable management option [23]. While food allergy immunotherapy is generally considered an experimental treatment, with many ongoing studies investigating different routes of administration, dosing regimens, as well as efficacy and safety [24,25,26], one oral immunotherapy for peanut allergy has been approved by Food and Drug Administration (FDA) [27]. Proper education is an important factor in food allergy management in order to guide patients’ attention to food labelling and their correct interpretation [28,29], raise awareness of possible cross-reactions with other food products [29,30], as well as for patients to know when and how to use prescribed medication [31]. Food allergy may have a negative impact on life quality [32], especially for kids who report decreased quality of social life and increased anxiety [33,34].

**Figure 1:**
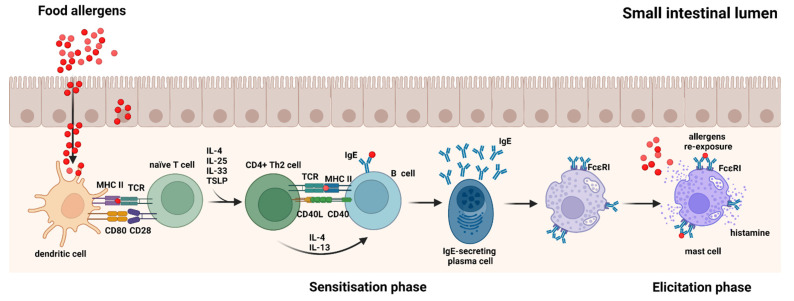
Mechanisms of IgE-mediated food allergy. IgE-mediated food allergy is divided into two phases: a sensitisation and an elicitation phase. In the sensitisation phase, food allergens are taken up by dendritic cells, which process allergens into smaller peptides and present them on MHC II molecules to T-cell receptors (TCRs) on naïve T cells. T cells are activated upon ligation of CD28 on the surface of naïve T cells and CD80 on the surface of dendritic cells, with co-stimulation from pro-inflammatory cytokines IL-4, IL-25, IL-33, and TSLP. Activated and differentiated Th2 cells interact and activate naïve B cells through TCR and antigen bound to MHC II on naïve B cells as well as through ligation of CD40L on the surface of Th2 cells and CD40 on the surface of B cells, together with co-stimulation from IL-4 and IL-13 for maturation and differentiation of B cells into food allergen-specific IgE-secreting plasma cells. Secreted food allergen-specific IgEs bind to high-affinity FcԑRI receptors on tissue mast cells and/or blood basophils. In the elicitation phase, re-exposure to the same or cross-reactive food allergens causes allergen cross-linking of FcԑRI-bound specific IgEs on the surface of tissue mast cells and/or blood basophils leading to their degranulation and release of mediators, such as histamine. Graphics created with BioRender.com.

Based on the mechanisms behind food allergy, the disease can be classified as either IgE-mediated or non-IgE-mediated allergy [35]. IgE-mediated food allergy is the best known and characterised type of food allergy [35,36] and can be divided into two phases: a sensitisation phase and an elicitation phase [37]. Upon a first exposure to food proteins, sensitisation may occur, when the immune system is introduced to the antigens for the first time. Antigen presenting cells (APCs), mostly dendritic cells (DCs), take up the food proteins or fragments hereof and process them into smaller peptides, which they present on their surface MHC II molecules to T-cell receptors (TCRs) on naïve T cells specific for the particular peptide. T cells are activated upon further signalling events with ligation of CD28 on the naïve T cells with CD80 and CD86 expressed on the surface of DCs as well as with co-stimulatory signals from pro-inflammatory cytokines IL-4, IL-25, IL-33, and TSLP [38,39], which causes the naïve T cells to differentiate into CD4+ Th2 cells [40,41]. Activated and differentiated Th2 cells interact with naïve B cells through their TCRs and allergen bound to MHC II on naïve B cells as well as through signalling events provided by binding of CD40L on the Th2 cells with CD40 on the B cells. This together with co-stimulatory signals from IL-4 and IL-13, secreted by Th2 cells, cause the B cells to maturate and differentiate into food allergen-specific IgE-secreting plasma cells [37,42]. Secreted food allergen-specific IgE binds to the high-affinity FcԑRI receptors on the surface of tissue mast cells or blood basophils [37], which completes the sensitisation phase (Figure 1). The elicitation phase takes place upon subsequent exposures to the same or cross-reactive food allergens, where the allergens cross-link FcԑRI-bound allergen-specific IgEs on the surface of the mast cells and basophils leading to their degranulation and release of mediators, such as histamine [37] (Figure 1). These mediators are responsible for the symptoms characterising the food allergic reaction, which can involve many organs causing, e.g., gastrointestinal disorders, respiratory tract inflammation, skin and eye itching and swelling, and in worst cases, life-threatening anaphylaxis [13].

### 2.1. Cow’s Milk Allergy

IgE-mediated CMA is the most common food allergy among infants and small children, affecting between 0.5 and 3.8% of the children [15,43,44]. Fortunately, most children outgrow their CMA, acquiring tolerance to cow’s milk [45], though some keep it for life [18]. CMA is usually one of the first food allergies diagnosed in infants, as cow’s milk proteins are often the first food proteins introduced to infants due to their presence in infant formulas [46]. Symptoms of IgE-mediated CMA most often appear immediately, within few minutes after consumption of the cow’s milk-based dairy product [47], and may reveal as diarrhoea, vomiting, skin itching, urticaria, or breathing problems, and may potentially cause anaphylaxis that can be fatal [48].

Little is known on why some individuals develop tolerance after consumption of cow’s milk proteins, while others develop an abnormal immune response towards the proteins. However, CMA can to some degree be “inherited”, as the atopy status of the child’s parents and siblings may be predictive for the risk of developing CMA [49].

Cow’s milk contains ~32 g of proteins per litre [50], which are divided into two protein fractions: caseins that represent ~80% and whey proteins that represent ~20% of the total proteins (Table 1) [51,52].

Cow’s milk allergens are designated Bos d, based on the three first letters of the genus and the first letter of the species epithet (*Bos domesticus*), followed by an identification number [55]. Bos d 8 is the allergen name registered in AllergenNomenclature covering all caseins [56]. However, as caseins are divided into four distinct types, they also have specific allergen names, with Bos d 9 designating α_s1_-casein, Bos d 10 designating α_s2_-casein, Bos d 11 designating β-casein, and Bos d 12 designating κ-casein. α_s1_-casein is the most abundant casein found in cow’s milk, comprising ~32%, followed by β-casein comprising ~28%, α_s2_-casein comprising ~10%, and κ-casein comprising ~10% (Table 1). They are classified as secreted calcium-binding phosphoproteins [57] with a loose tertiary structure. In their soluble form, they create quaternary structures called casein micelles. Casein micelles contain a hydrophobic core consisting of α_s1_-casein, α_s2_-casein, and β-casein interacting with calcium phosphate and a hydrophilic surface layer of κ-casein [58]. In general, the casein micelle structure is dynamic and changes with factors such as pH, temperature, and pressure. For example, under rennet treatment, casein micelles lose their solubility and precipitate forming aggregates [59], and at various temperatures, micelles may form numerous interactions to a different extent with whey proteins and other milk components [60]. Caseins are all major allergens considered to be involved in more than 50% of all IgE-mediated CMA reported cases [61].

The most abundant whey protein is β-lactoglobulin, designated Bos d 5, which represents ~10% of total proteins in cow’s milk and is followed by α-lactalbumin, designated Bos d 4, comprising ~5%; immunoglobulins, designated Bos d 7, comprising ~3%; bovine serum albumin, designated Bos d 6, comprising ~1%; and lactoferrin, comprising <1% (Table 1). β-lactoglobulin and α-lactalbumin are considered major allergens from the whey fraction. They are globular proteins, stabilised by disulphide bridges [62]. Even though bovine serum albumin is found in cow’s milk in only low quantities, it is also a common allergen, as up to 50% of cow’s milk allergic patients develop IgE specific for this protein [63,64]. Together with lactoferrin, bovine serum albumin is characterised by a high number of disulphide bridges (Table 1), making their tertiary structure highly stable even under denaturing conditions [64]. Lactoferrin is a protein not registered as an allergen in the AllergenNomenclature [56], however, human and animal experimental studies showed their ability to induce allergic reactions [65,66,67].

Generally, it is not so common that cow’s milk allergic patients react to only one cow’s milk protein, as CMA is usually characterised by reactivity to multiple cow’s milk allergens, including both the unstructured caseins and the globular whey proteins [67]. There are specific sites within the protein sequence and/or structure that IgEs bind to, which are called epitopes [68]. These epitopes can be either linear or conformational, with linear epitopes consisting of a continuous amino acid sequence of the primary protein structure and conformational epitopes consisting of discontinuous amino acid sequences brought together by the secondary, tertiary, and quaternary folding of the protein [68,69]. Both types of epitopes are found in cow’s milk allergens [37,70].

### 2.2. Prevention and Management of Cow’s Milk Allergy

To prevent the development of CMA in high-risk infants as well as to manage CMA to avoid elicitation of allergic reactions in already allergic infants, guidelines have been devised providing specific recommendation. Yet, recommendations for CMA prevention and management have changed during the past decade, as recent studies have provided new knowledge with further evidence on for example maternal elimination diet [71], vaginal birth versus caesarean [72,73], pre- and probiotics supplementation [74,75], duration of breastfeeding [76], time of introduction of allergenic foods [77,78,79], and use of hydrolysed infant formulas [76,80] in relation to CMA prevention and management.

The European Academy of Allergy and Clinical Immunology (EAACI) guidelines on CMA prevention published in 2014 and 2021 concluded that there is no need for maternal elimination diet during pregnancy as well as during lactation period, as the majority of trials have shown no relationship between maternal elimination diet and a reduction in the probability of CMA occurrence in offspring [23,81]. These conclusions provided by the EAACI guidelines for CMA prevention are in line with the Australasian Society of Clinical Immunology and Allergy (ASCIA) guidelines for food allergy prevention from 2005 and 2019 [82,83] as well as with the guideline by the American Academy of Allergy, Asthma, and Immunology (AAAAI) on CMA prevention from 2021 [84]. In addition, a Cochrane Systemic Review by Kramer and Karkuma based on five trials concluded that there is no relation between elimination diet during pregnancy and lactation and a lower likelihood of events of atopic diseases [85]. On the other hand, a cohort study by Tuokkola et al. showed that consumption of cow’s milk proteins during pregnancy and lactation contributed to a lower risk of CMA development in offspring compared to those whose mothers avoided the consumption of cow’s milk proteins during pregnancy and lactation [86]. This is in line with a study by Stravik et al., which showed similar results [87].

Transmission of the maternal microbiome during vaginal birth is a very important and beneficial factor influencing later gut microbiota development in the infant [88]. The first 1000 days of a child’s life is crucial for lifelong gut microbiota shaping [89]. Gut microbiota composition is known to have an influence on many health aspects, including probability of development of many diseases [90]. In relation to CMA prevention, the evidence is contradictory, as some studies have shown no relationship between caesarean delivery and thus the lack of maternal vaginal microbiome transmission and an increased risk of developing allergy [73,91], while others reported such relationship, indicating a beneficial impact of maternal microbiome transmission during vaginal birth for prevention of CMA in offspring [92,93].

Supplementation of probiotics may in some situations be beneficial for an infant. This has, for example, been shown during antibiotic treatment, where the gut microbiota can be heavily disrupted [94]. However, from the perspective of CMA prevention, there is no evidence for or against the use of probiotics in both infants as well as in the breastfeeding mothers [95,96]. For prebiotics, such as galacto-oligosasccharides (GOS) and fructo-oligosaccharides (FOS), which are used for infants to promote a healthy gut microbiota, there is also no evidence for or against their use in the prevention of CMA [75].

In relation to breastfeeding as a potential factor in preventing CMA, current evidence shows no relation between breastfeeding and lower risk of CMA development. However, breastfeeding is anyway strongly recommended by EAACI [81], ASCIA [83], and AAAAI [84] guidelines, as it has many benefits for both infant and mother. Therefore, it should always be the first choice for infant feeding, as mother’s milk has the best nutritional composition designed to meet the infant’s need, as it changes continuously, adapting to infants’ specific age and hence growth need. Furthermore, the World Health Organisation (WHO) strongly recommends exclusive breastfeeding for the first 6 months of life and thereafter continued breastfeeding while introducing complementary food for as long as the child and mother are willing to [97].

Delayed introduction of the most common allergens by time of complementary food introduction for prevention of food allergy, including CMA, is strongly discouraged by EAACI, ASCIA, and AAAAI, as there is no evidences for its beneficial effect [81,83,84]. In fact, several studies have shown that early introduction of allergenic foods, such as peanuts [98,99], cow’s milk [100,101], or hen’s egg [99], can decrease the risk of developing food allergy against the particular allergens [102].

Recommendations on the use of special infant formulas based on hydrolysed cow’s milk proteins for CMA prevention have changed over the past years. Until recently, it was recommended to use a cow’s milk-based partially hydrolysed formula (pHF) for infants in high-risk of developing CMA [23,82]. However, as the current evidence shows no relationship between the use of pHF and a decreased risk of developing CMA [103], the recent guidelines from EAACI, ASCIA, and AAAAI do not recommend using pHF or any other specific infant formulas for CMA prevention [81,83,84].

There are several guidelines with recommendations for CMA management available. The European Society for Paediatric Gastroenterology Hepatology and Nutrition (ESPGHAN) [104], British Society for Allergy and Clinical Immunology (BSACI) [105], as well as World Allergy Organisation (WAO) [106] created practical guidelines for CMA diagnosis and management, which are consistent. For the management of CMA in infants, breastfeeding along with strict avoidance of intact cow’s milk proteins are the first strategies recommended [104,105,106,107]. If breastfeeding is impossible or insufficient, the use of a hypoallergenic infant formula is recommended with eHF as a first choice [104,105,108], where a hypoallergenic infant formula is required to be tolerated by at least 90% of the infants with confirmed CMA, with a confidence interval of 95% in a clinical cohort [80]. If clinical symptoms occur with the use of eHF, the use of AAF is the recommended as the second choice for CMA management [104,105,108].

It has been reported that ~0.5% of infants exclusively breastfed develop cow’s milk allergic reactions though mostly reported as being mild or moderate [108]. This may be due to low quantities of cow’s milk proteins being present in breastmilk after the consumption of dairy products by the mother [109]. Thus, the maternal diet while breastfeeding an infant diagnosed with CMA needs to be monitored by physician, and in most cases, elimination of any products containing cow’s milk proteins is recommended for the mother [104,110]. Another important factor for CMA management is awareness of possible cross-reactivity between cow’s milk proteins and proteins from other mammalian milks. In addition, proteins found in cow’s milk can also be found elsewhere than in milk as, for example, serum albumin, a whey protein, is also present in beef meat as well as in cow’s dander [111].

## 3. Infant Formulas

Breastfeeding is not always a possibility, as it may be insufficient or not chosen for several reasons. Hence, an alternative to breastfeeding is needed.

Infant formulas are substitutes to breast milk, manufactured in order to fulfil the nutritional requirements of infants allowing their ordinary growth [112,113]. They should mimic breast milk, providing similar conditions for infants’ development before and during the introduction of complementary food, until the complete transition to solid food [51]. Indeed, in EU, infant formulas are strictly regulated and need to comply with the Regulation EU 2016/127 with regards to specific compositional and informational requirements [114]. This EU legislation incorporates the principles from WHO Code of Breastmilk Substitutes [115]. If an infant is not breastfed, formulas should be the main source of nutrition for the infant up to 12 months of age. In the EU, the only sources of protein allowed in infant and follow-up formulas are cow’s milk, goat milk, soy, as well as hydrolysed proteins [7]. Yet, the major part of infant formulas are based on cow’s milk proteins and should not be confused with any unmodified, raw, or pasteurised milk commercially available [116], as these are not able to fulfil the nutritional requirements of the infants [5,117]. Infant formulas, in general, contain a higher amount of protein compared to breast milk but a lower amount of protein compared to regular cow’s milk [5]. In addition, the protein composition may differ, with the proteins in soy-based formulas being very different from those in breast milk [118], and where, for example, the ratio of casein to whey proteins present in cow’s milk-based infant formulas may differ from the ratio in breast milk as well as in regular cow’s milk, which may influence the properties of infant formulas, including their digestibility [5,119]. A slower digestion kinetics of casein-dominant infant formulas compared to whey-dominant formulas have been shown using an in vitro dynamic infant gastric simulator, which might be explained by a greater extent of aggregations in the casein-based formulas [120].

The lipid content in infant formulas is designed to mimic the composition and amount in breast milk and consists of long-chain polyunsaturated fatty acids (LCPUFAs), such as eicosapentaenoic acid (EPA) [121] and docosahexaenoic acid (DHA), for proper brain development [122] as well as arachidonic acid (ARA) for proper nervous system and muscles development [123]. As the lipid composition in infant formulas should mimic the composition in human milk as much as possible [124], human milk oligosaccharides (HMOs) have gained an increasing interest in the recent decade, especially due to their important impact on the development of a healthy gut microbiota and immune system [125]. GOS and FOS are often included in infant formulas as prebiotics for proper intestinal microbiota development [126]. Iron is an important mineral for a proper neurodevelopment, for which reason, in contrast to regular cow’s milk, infant formulas are fortified with iron [127,128].

## 4. Cow’s Milk-Based Infant Formulas

Most infant formulas commercially available are based on cow’s milk proteins [51] due to the great availability of the dairy cow’s milk worldwide, which corresponds to 81% of the worlds’ milk production [129]. In this review, “infant formula” refers to cow’s milk-based infant formulas unless stated otherwise. In general, infant formula manufacture is based on milk reconstitution, where different milk fractions, including proteins (whey proteins and/or caseins), fat, and micro- and macronutrients together with other non-milk-based ingredients, are mixed together in specific quantities to fulfil infant formula standards and nutritional requirements in accordance with Regulation EU 2016/127 [114].

**Figure 2:**
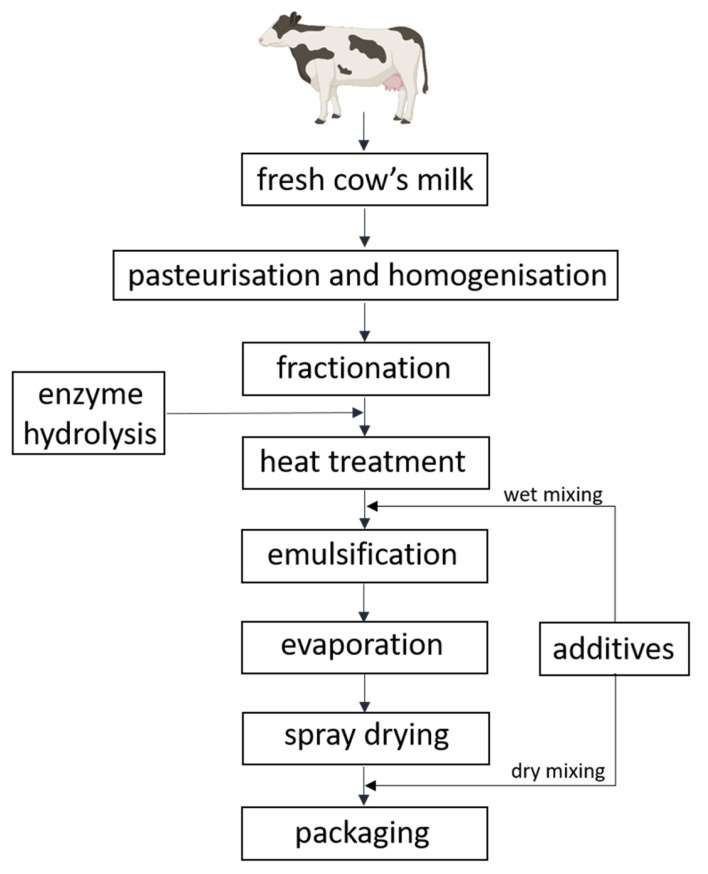
Example of main steps involved in the manufacturing of powdered infant formula. Fresh cow’s milk is at first standardised by means of pasteurisation and homogenisation. Further, milk proteins are going through fractionation, heat treatment, mixing, emulsification, evaporation, spray drying, and packaging. Additives are added and mixed with the protein fraction by means of wet or dry mixing. If wet mixing is applied, additives are added in a liquid form to a liquid protein fraction and together undergo emulsification, evaporation, spray drying, and finally infant formula powder packaging. If dry mixing is applied, the same steps are applied with the exception of additives being mixed with proteins in powdered form after spray drying. Additionally, if an infant formula is produced to be used in CMA management, enzyme hydrolysis of proteins is applied after the fractionation step. Picture of the cow is from BioRender.com.

Infant formulas can be sold as powder, liquid concentrate, or ready-to-use liquid, where powder-based infant formulas are the cheapest and most common [130]. Figure 2 displays infant formula powder production steps, where technological processes, including pasteurisation, homogenisation, fractionation, heat treatment, mixing, emulsification, evaporation, spray drying, and packaging, are used [51,130,131]. Infant formulas can be based on the whey or casein fraction or both [132]. If both fractions are used in infant formula, fractionation is still applied for whey and caseins separation for their further ratio adjustment (Figure 2). More infant formulas based on whey proteins than caseins are available due to the focus on optimal utilisation of whey after cheese production [133]. There are two methods for mixing additives with milk proteins that can be applied during powdered infant formula manufacture: wet or dry mixing (Figure 2) [130]. In the wet mixing method, additives in liquid form are added to liquid milk proteins, and subsequently, they are spray dried together, whereas in the dry mixing method, additives are added after spray drying to powdered milk proteins, and powders are mixed together. Infant formulas, where no additional processing steps are applied for intentional induction of changes in protein structures, are known as conventional infant formulas. These formulas, based on intact cow’s milk proteins, are the most widely applied formulas but are not recommended for infants with CMA [106,134]. Infant formulas that are produced to be used for CMA management are based on enzyme hydrolysed cow’s milk proteins, where enzyme hydrolysis is applied after protein fractionation (Figure 2) [135].

### 4.1. Reduction of Cow’s Milk Protein Allergenicity by Process Modifications

The most common alternatives to conventional infant formulas are infant formulas based on cow’s milk proteins, where the proteins are altered to a degree that allows a decrease in their allergenicity, still keeping their nutritional, functional, and palatable properties [136].

**Figure 3:**
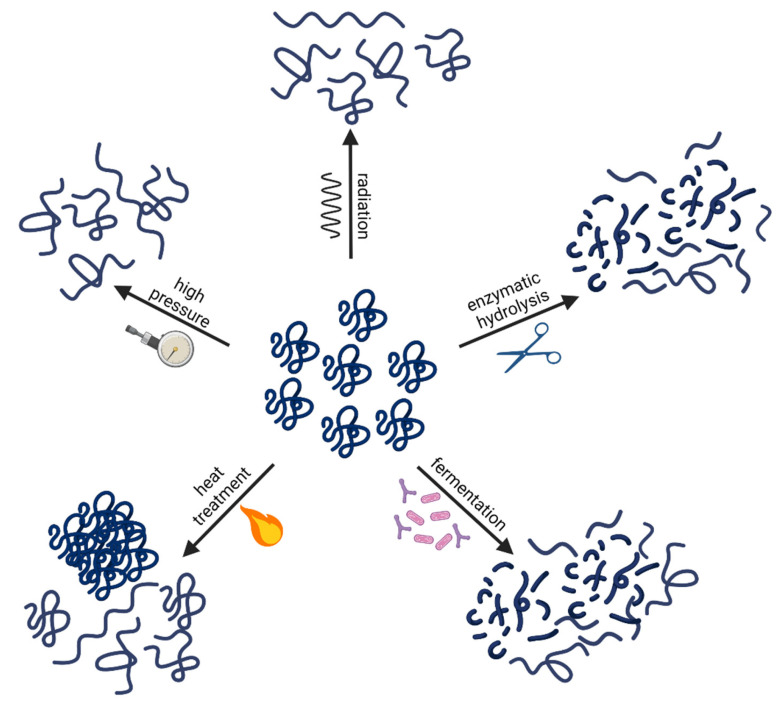
Common protein structural modifications induced by different processing technologies. Enzyme hydrolysis and fermentation may lead to proteolysis of the proteins, destroying primary, secondary, tertiary, as well as potential quaternary structures and causing proteins to break down to smaller peptides. Heat treatment may cause protein denaturation and/or aggregation, while high pressure and radiation may cause protein denaturation. Graphics created with BioRender.com.

Alteration and hence potential reduction of cow’s milk protein allergenicity may, in general, be induced by several processing technologies, such as enzymatic hydrolysis, fermentation, heat treatment, high pressure (HP), and radiation (Figure 3). The overall aim of such processing is to diminish or even destroy the IgE-binding epitopes in order to avoid de novo sensitisation in an infant not previously exposed to cow’s milk proteins or to avoid cross-linking of IgEs on the surface of tissue mast cells and blood basophils, averting degranulation and hence elicitation of an allergic response in CMA infants [134,137]. Reduction and/or destruction of the IgE-binding epitopes are caused by protein aggregation, denaturation, and degradation. Hence, it is also well recognised that general cooking alters food protein allergenicity by changing, masking, or even destroying IgE-binding epitopes [134].

#### 4.1.1. Enzymatic Hydrolysis

Enzyme hydrolysis is the most common process used in the infant formula industry to induce protein modifications. The main purpose of this process is to break linear as well as conformational epitopes, hence destroying primary, secondary, tertiary, as well as potential quaternary protein structures (Figure 3) [8]. Enzyme hydrolysis can result in different degree of hydrolysis (DH) depending on the number of enzymes used, their specificity, as well as the conditions applied, such as pH, duration, and temperature. The most common enzymes used in the infant formula industry are recombinant, non-porcine-based proteases for final product Kosher and Halal status [138]. There is a great variation in susceptibility to hydrolysis between different cow’s milk proteins depending on their structure and the enzyme(s) used. In general, whey proteins, and especially β-lactoglobulin, are known to be more resistant to proteolysis than caseins [53]. This is explained by the globular structure of the whey proteins that is stabilised by a number of disulphide bridges (Table 1), making it difficult for enzymes to access their cleavage sites. It is, however, shown that susceptibility of proteins to proteolysis can be increased with a preceding heat treatment for protein unfolding [139].

Infant formulas based on hydrolysed cow’s milk proteins can be classified as either eHF or pHF depending on the sizes of the peptides in the final product relating to the DH [140]. There is no uniform definition of eHF and pHF [80,141], but in general, eHFs predominantly contain peptides of sizes below 3 kilodalton (kDa), whereas pHFs predominantly contain peptides of sizes below 5 kDa although larger peptides may appear [80,141]. However, great variations exist between different products, and even significant product batch-to-batch variations have been demonstrated [8,132].

In general, there is no universal definition of a hypoallergenic infant formula. Yet, infants with confirmed CMA are recommended the use of a hypoallergenic infant formula, where the allergenicity of proteins are reduced in order to avoid elicitation of allergic reactions, thus suitable for the management of CMA [104]. The American Academy of Pediatrics (AAP) described requirements that an infant formula needs to fulfil in order to be used for CMA management, thus having “hypoallergenic” status [80]. The hypoallergenic infant formulas should be tolerated by at least 90% of the infants with confirmed allergy to cow’s milk proteins with a confidence interval of 95% based on clinical trials [6,80,135]. eHFs are produced to meet these requirements, and according to ESPHGAN [104], all peptides in eHFs should have a size < 3 kDa and be dominated by peptides with a size ~1.5 kDa, hence containing at maximum one linear epitope and thus should not be able to cross-link IgEs on the surface of tissue mast cells and blood basophils and cause allergic reactions [1,142]. eHFs are recommended as a first choice for infant CMA management when breastfeeding is insufficient, impossible, or simply not chosen [104,143]. Yet, some infants with CMA experience allergic reactions, even anaphylaxis, upon feeding with eHFs [104] and consequently may rely on AAF (see Section 5) or if available a soy- or hydrolysed rice-based formula [144] (see Section 7.1 and Section 7.2).

pHFs are characterised by their reduced allergenicity compared to conventional infant formulas [145], providing them with decreased potency to induce de novo sensitisation. Yet, they still contain peptides large enough to be recognised by the immune system for induction of tolerance, hence maintaining the tolerogenic properties [141]. However, infant formulas containing peptides between 3–5 kDa, thus being composed of 22–36 amino acids, may induce an allergic reaction, as the peptides could potentially contain two IgE-binding epitopes, allowing for cross-linking of IgEs on the surface of tissue mast cells or blood basophils [1].

Allergenicity as well as eliciting capacity of pHF were evaluated in human studies. For example, Niggemann el al. showed a reduced allergenicity of pHF in patients with CMA as well as reduced eliciting capacity based on skin prick test (SPT) [146], while a study by Caffarelli et al. showed reduced allergenicity and reduced eliciting capacity of some pHFs but not of other pHFs when compared to cow’s milk [147]. Moreover, there are animal models established for the evaluation of inherent immunogenicity and allergenicity of infant formulas as well as for the assessment of their preventive capacity [148,149,150,151,152]. Several animal studies have shown reduced allergenicity of pHF [153,154,155]. In one study, it was shown that pHFs did not induce sensitisation [155], while another study showed induction of sensitisation but without clinical symptom manifestation [156].

Several human studies have been conducted for evaluation of the preventive effect of pHF on CMA development, showing different outcomes. Whereas Vandenplas et al., Chandra, and van Berg et al. provided evidence for a preventive effect of pHF on CMA development when comparing with conventional infant formula [157,158,159], Lowe et al. did not find evidence to support use of pHF in CMA prevention in comparison to conventional infant formula [160]. In agreement with Lowe et al., a systemic review by de Silva et al. concluded that neither the use of hydrolysed infant formula nor avoidance of conventional infant formula had an effect on CMA prevention [161]. Results from animal studies are in line with the results from human studies, with some studies showing a preventive effect of pHFs on CMA development, while other studies did not show such effect. For example, studies by Graversen et al., Jensen et al., and Fritsche et al. showed that partially hydrolysed whey had a capacity to induce oral tolerance to intact whey proteins [151,162,163]. Further, Chikhi et al. showed that partially hydrolysed whey induced a partial prevention of sensitisation to β-lactoglobulin but no prevention of sensitisation to caseins [164].

Differences in the results from both animal and human studies in relation to the preventive effect of pHF on CMA development may be a result of the large variation in pHFs characteristics. Different pHFs characteristics may be explained by whether the pHFs are exclusively based on whey proteins or caseins or on whole milk. For example, lack of the preventive effect of partially hydrolysed whey on casein sensitisation development may be explained by the lack of casein derived peptides in this type of product and, as a consequence, lack of the oral tolerance induction towards caseins as explained in the study by Chikhi et al. [164]. In addition, different pHFs characteristics may be explained by the huge variation in DH, as illustrated by Graversen et al. [162].

Whereas the EAACI guideline from 2014 recommended the use of pHF for prevention of CMA [23], the recent EAACI guideline from 2021 has been updated and no longer provides specific recommendation for use of pHF [81] due to the lack of evidence for superior effect of pHF in preventing CMA. In line, the AAAAI [84] and ASCIA [83] guidelines likewise concluded that there is no evidence for recommending either against or for the specific use of pHF in CMA prevention. Guidelines in general emphasise the importance of considering each infant at high-risk of developing CMA independently, giving recommendations on infant formula based on their own individual circumstances [81,83,84].

Currently, there are no universal criteria for eHF production or batch-to-batch variance control, and different companies apply procedures with different enzyme hydrolysis conditions for their product manufacture, resulting in varying DH as well as varying peptides size distribution profiles [135,165]. Moreover, many producers do not have published data of their product safety and efficacy, including peptide profile and their residual immunogenicity and allergenicity [8,166]. Therefore, there is a need for uniform pre-clinical in vivo and in vitro testing procedures for evaluating residual allergenicity of future hypoallergenic infant formulas [153]. Variations in eHF characteristics result in different outcomes of their evaluation as a CMA management option both in human as well as animal studies.

eHFs are well tolerated by most cow’s milk allergic infants. This is supported by animal studies showing that eHFs are suitable for CMA management, as they, in general, lack inherent allergenicity and do not induce clinical symptoms in cow’s milk allergic animals [153,167]. Yet, several human studies have shown reactivity towards eHFs in some cow’s milk allergic infants due to residual allergenicity still present even after extensive hydrolysis [8,166,168,169], indicating that eHF cannot be used for CMA management in all cow’s milk-allergic infants.

Although eHFs are the best suited infant formulas for use in infants suffering from CMA who are not fully breastfed [104], they may, in addition to not being tolerated [144], be refused by some infants, as they may have a bitter taste due to hydrophobic amino acids that are exposed after hydrolysis [166,170,171], or be regarded as too expensive [1].

#### 4.1.2. Fermentation

The ESPGHAN defines fermented formulas as infant and follow-up formulas that have been fermented with lactic acid producing bacteria during the production process but do not contain significant amounts of viable bacteria in the final product due to inactivation of the fermenting bacteria by for example heat treatment [104]. Hence, they are different from prebiotic or probiotic products in that they lack viable bacteria or prebiotic oligosaccharides but contain fermentation products, which might modulate gut immunity or gut microbiota, and promote allergy prevention [172]. Proteolytic enzymes secreted by lactic acid bacteria break down milk proteins, as displayed on Figure 3, leading to the degradation of IgE epitopes [137]. Indeed, peptides from the proteolysis of β-lactoglobulin and α-lactalbumin have been detected after fermentation of whey proteins by *Lactobacillus* species [173]. Destruction of β-lactoglobulin and casein epitopes could explain the reduction in binding of IgE from cow’s milk allergic children to these proteins, as observed in several studies [174,175,176]. Infant formulas fermented by other bacteria than *Lactobacillus* species (e.g., *Bifidobacterium*) have also been investigated and have shown a capacity to strengthen the intestinal barrier in mice [177]. Moreover, a systematic review on the health benefits of fermented infant formulas concluded that there was evidence of reduced incidences of respiratory (e.g., bronchitis, wheezing) and gastrointestinal (e.g., vomiting, diarrhoea, colitis) allergic reactions in cow’s milk allergic infants [178,179]. However, there is not yet enough supporting evidence for the use of fermented formula for prevention or management of CMA [178,179], and more information on the exact composition and molecular structure of the fermented products as well as in-depth knowledge of mechanism of fermentation are needed for the optimisation of fermented infant formulas. Currently, no fermented infant formulas are commercially available.

#### 4.1.3. Heat Treatment

Heat treatment of infant formulas or infant formula ingredients is used during the processing of these products to ensure microbiological safety and to obtain a long shelf life but not specifically to reduce milk allergenicity [133,134]. Pasteurisation (82 °C for 15 s or 94 °C for 30 s), in-can sterilisation (>110 °C for 10−30 min), spray drying (150–200 °C), or ultra-high temperature (UHT) treatment (135−150 °C for 2−6 s) are the most common heat treatments applied, and in some cases, they are combined [180,181]. However, information on the exact heating conditions is usually not available, as this information is commercially sensitive. Heating may induce modifications of amino acids in proteins, leading to changes in the protein structure and promoting interactions between proteins as well as between proteins and other ingredients in the infant formula (Figure 3) [182,183]. These modifications may affect, for instance, protein bioavailability, digestibility, as well as the presence and/or accessibility of IgE-binding epitopes and hence protein allergenicity [184,185,186]. The extent of the heat-induced alterations will be determined by the differences in and combination of processing, dependent on time, temperature, and rate of heating, as well as the composition of the infant formula.

Caseins lack well-defined secondary or tertiary structures, which render them very stable to high temperatures. Yet, heat treatment can lead to their precipitation and aggregation [187,188,189,190]. Whey proteins are in contrast generally susceptible to heat treatment and might undergo irreversible denaturation and aggregation as well as interact with casein micelles resulting in decreased solubility of the proteins [60,191,192,193]. Whereas β-lactoglobulin, the most abundant protein in whey, unfolds and aggregates at temperatures > 65 °C, α-lactalbumin is a bit more heat resistant, unfolding at temperatures > 70 °C, however, without formation of aggregates [184,190,194,195,196,197].

It has been reported that infant formulas are less heat stable than regular cow’s milk, and whereas changes in protein secondary structure in the infant formula have been shown to begin at 50 °C, substantial changes in regular cow’s milk were observed at 70 °C [198]. Changing the protein composition of infant formulas has significant effects on the formula heat stability. For example, in a study by Crowley et al., it was shown that increasing the ratio between α-lactalbumin and β-lactoglobulin increased the heat stability of the infant formula [199], which is in agreement with a study by Halabi et al., who observed that infant formulas with high α-lactalbumin and lactoferrin content were protected against heat denaturation of native whey proteins [200].

Generally, heat-induced denaturation caused by intense thermal processing promotes digestion of milk proteins [186,201,202]. For instance, digestion of caseins in infant formulas heated at 80 °C was faster than the digestion of the unheated counterpart, which could be explained by their smaller micelles covered by denatured whey protein aggregates, thus increasing the accessibility to proteases [203]. Further, another study showed that upon more intense temperatures of 120 °C, caseins were even more rapidly digested than after pasteurisation at 82 °C [204]. Likewise, several studies have shown that heating at temperatures between 75 and 90 °C denatures β-lactoglobulin and increases its accessibility to proteases and thus its digestibility [139,186,205,206].

Exposure to high temperatures during processing of infant formulas or infant formula ingredients can result in protein oxidation, where sulphur-containing amino acids as wells as aromatic amino acids are particularly susceptible to oxidation [207,208,209,210,211,212]. These modifications result in aggregation via covalent cross-linking or hydrophobic interactions as well as alteration of amino acids and protein conformation [213,214]. The extent of protein modifications seems to depend on the heating conditions. Moreover, oxidation-based modifications, such as formation of dityrosine, increase surface hydrophobicity and viscosity and might be responsible for the reduced digestion of infant formulas [215,216]. Oxidation seems to be higher in infant formula compared to regular cow’s milk [217].

In addition to oxidation, heat-induced glycation reactions between proteins and sugars in infant formula or infant formula ingredients might occur [181,218,219,220,221]. More specifically, interactions between the amino groups in proteins and reducing sugars, such as glucose or lactose in the milk, result in Maillard reaction products called Amadori products, which can undergo further reactions resulting in advanced glycation end products, such as carboxymethyl-lysine [181]. The glycation of lysine residues protects amino acids from proteolysis, decreasing protease accessibility, thus impairing protein digestion [182,186,222,223]. There is not uniformity in the degree of heat-induced modifications among similar infant formula ingredients from different manufacturers [224]. Generally, the degree of modifications and the number of modified proteins increase with higher temperatures and/or longer heating durations [221,225,226,227], and thermal processing during infant formula production has been shown to increase the presence of Maillard products compared to regular cow’s milk [181,218]. Maillard products are not only present in the final infant formulas but already in the infant formula protein ingredients, as pasteurisation, emulsification, evaporation, spray drying, and sterilisation (Figure 2) of both whey and casein fractions may give rise to Amadori products [228,229].

The effect of heat treatment on allergenicity of cow’s milk has been extensive studied, but only few reports have been described using infant formulas or infant formula ingredients [64,134,230,231]. In general, heat processing may have an impact on infant formula allergenicity either as a direct effect conferred by the protein modifications induced or as an indirect consequence of altered bioavailability and digestibility. Consequently, using optimised heat treatment as a processing method, infant formulas with low allergenicity could be produced. The molecular basis of modifying allergenicity is the destruction/masking of the IgE epitopes and/or exposure/formation of new epitopes by denaturation, aggregation, and amino acid modifications, thus reducing or enhancing IgE recognition [64]. Accessibility to β-lactoglobulin epitopes is temperature dependent. Temperatures < 90 °C increase β-lactoglobulin antigenicity due to protein unfolding and exposition of epitopes buried inside the native molecule; however, heating > 90 °C induces aggregation and amino acid modification, masking or destroying conformational and linear epitopes and thus decreasing both its antigenicity and allergenicity [231,232,233,234,235]. Contrary to whey proteins, caseins are thermostable and thus retain allergenicity even after extensive heat treatment [235].

The degree, length, and rate of heating; the type and concentration of reducing sugars; and the extent of glycation could be adjusted in order to influence allergenicity of infant formulas. In one study, conjugation of whey protein with maltose was shown to be an effective way to reduce the antigenicity of α-lactalbumin and β-lactoglobulin [230]. Another study showed that moderate glycation did only have a small effect on binding of IgE from cow’s milk allergic patients to β-lactoglobulin, whereas a high degree of glycation masked IgE epitopes, reducing the recognition by IgE from allergic individuals [236].

Heat treatment affects allergenicity not only by modifying epitope recognition but also by hindering protein uptake and changing the uptake route. Indeed, heat-induced aggregation of whey proteins during pasteurisation impaired protein uptake through epithelial cells in a mouse model and thus protected against an allergic response [237]. In another study, Graversen et al. showed that partial heat-induced protein denaturation and aggregation of whey proteins changed the proteins route of uptake, being more efficiently transported through Peyer’s patches, which might explain the reduced allergenicity of the modified whey proteins [238].

In general, studies considering the effect of heating on allergenicity indicated that the effect is very complex and dependent on many factors and not only on the heat stability and concentration of the proteins as well as the heat treatment regime but also on the presence of other components in the formula [134]. Yet, further research is needed to gather knowledge on how to alter the processing parameters applied to infant formulas and the specific formulation to produce safe products for cow’s milk allergenic individuals.

#### 4.1.4. High Pressure

Non-thermal processing has been investigated as a method for reducing allergenicity of cow’s milk proteins either as an alternative method to or in combination with thermal processing although only few studies have been performed with infant formula or infant formula ingredients [64,134]. One method investigated is HP treatment (200–600 MPa) although HP-based infant formulas are not commercially available yet. The HP process can affect non-covalent interactions, such as hydrophobic or electrostatic interactions between milk proteins, as well as affect the protein structure (Figure 3). Thus, HP-derived modifications may alter protein allergenicity. In fact, HP treatment (400 and 600 MPa) of whey proteins was shown to disrupt protein interactions and alter protein structure with resulting exposure of linear epitopes that were hidden in the native structure of the proteins [239]. Consequently, HP increases the allergenicity of whey proteins, depending on the exact time and degree of pressure, by increasing epitope accessibility and hence enhancing their allergenicity. Combination of HP and heat treatment (600 MPa, 40 °C) was shown to have a synergistic effect, which further increased the allergenicity of β-lactoglobulin [239,240]. On the contrary, studies performed by Chicón et al. showed that HP treatment (200 and 400 MPa) of whey proteins did not affect β-lactoglobulin allergenicity by means of binding to IgE [241].

A novel HP-based method that combines HP and short-term heat treatment, followed by an instant pressure drop to vacuum, has been investigated for reducing allergenicity of whey proteins and caseins. The protein conformational changes and aggregations observed resulted in opposite effects on the allergenicity for the whey and casein fractions, with a decreased allergenicity for whey proteins and an increased allergenicity for caseins [242].

#### 4.1.5. Radiation

Microwave, ionisation (e.g., X-ray, high-energy electron beams, or γ-rays), ultraviolet (UV), or infrared radiation have gained much attention in the last decade because they induce conformational changes and denaturation of milk proteins (Figure 3), leading to the alteration in their epitopes [134,243,244,245]. For example, it has been reported that the allergenicity of β-lactoglobulin decreased by γ-radiation [246] and that the allergenicity of α-caseins and whey proteins decreased by UV treatment [244]. Yet, the effect on infant formulas has not been assessed.

#### 4.1.6. Other Processing Technologies

Based on the literature available, it seems that other techniques, such as ultrasound and non-thermal atmospheric plasma, have no effect in reducing the allergenicity of whey proteins and caseins [247,248] although changes in the secondary structure of β-lactoglobulin were observed upon ultrasound application [247]. The impact of the processing techniques, pulse electric field, and ohmic heating on milk allergenicity has not been investigated yet but could be considered in the future, as it has been shown that a pulsed electric field induces structural modification of whey proteins [249].

## 5. Amino Acid-Based Infant Formulas

AAFs are exclusively based on free amino acids and are free from peptides derived from cow’s milk proteins. They are used in infants with severe CMA where eHF cannot resolve all symptoms or in those cases where anaphylaxis occurs [250]. However, in some regions of the world, there is an excessive use of AAF due to the lack of proper diagnostic tools as well as resources to perform subsequent oral food challenge (OFC) for evaluation of acquisition of tolerance to cow’s milk [251]. However, in EU, the use of AAF is only recommended if eHF cannot be used for CMA management.

Hypoallergenicity and thus the safety of AAF has been proven by several clinical trials showing that AAFs are well tolerated by infants suffering from severe CMA [252,253]. Nutritional aspects of AAF have also been assessed in order to evaluate whether infants fed with AAF have a normal growth rate when comparing with those fed with other types of infant formulas, which concluded that AAF supports a normal growth of infants [254,255,256].

## 6. Infant Formulas Based on Mammalian Milk Proteins

As previously stated in this review, cow’s milk is the main source of proteins in dairy product manufacturing, including production of infant formulas, due to its great availability [129]. However, there is an increasing interest in the utility of other mammalian milk as a source of proteins in infant formula manufacture. At present, in EU, only milk proteins from cows and goats are allowed to be used in infant formula production in accordance to the EU legislation [7,257]. For dairy product manufacture, regular milk from non-cattle species, such as *Capra hircus* (goat), *Ovis aries* (sheep), and *Camelus dromedaries* (camel), contributes with ~17% of the global milk production. Milk from *Equus asinus* (donkey) or *Equus ferus caballus* (horse) are also gaining an increased interest for dairy product manufacture though on a smaller scale compared to goat, sheep, and camel milk [129].

Due to the population growth and hence the increasing need for protein sources, there is a demand for more and new dairy products, including those based on non-cattle milk [258]. Cow’s milk is the most common source of proteins in infant formula both in the production of conventional and in the production of hydrolysed infant formulas [53]. The composition of mammalian milk differs between different animals and are different from breastmilk, with differences in total protein content, casein-to-whey protein ratio, protein composition, as well as differences in individual protein amino acid sequences.

**Figure 4:**
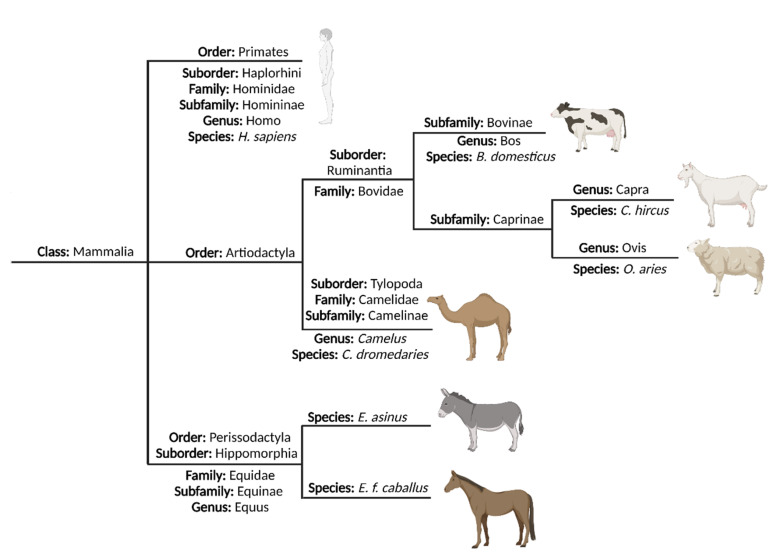
Relationship between present and potential future mammalian milk sources for infant formula manufacture. *B. Domesticus* (cow), *C. hirsus* (goat), *O. aries* (sheep), and *C. dromedaries* (camel) belong to the order Artiodactyla. Camel belongs to the suborder (Tylopoda), while cow, goat, and sheep belong to the same suborder (Ruminantia) as well as family (Bovidae), with cow belonging to Bovinae subfamily and goat and sheep belonging to Caprinae subfamily. Goat and sheep differ in their genius, where goat belongs to Capra, and sheep belongs to Ovis. Camels belong to Camelidae family, Camelinae subfamily, and genus *Camelus*. *E. asinus* (donkey), and *E.f. caballus* (horse) both belong to the order Perissodactyla as well as the same suborder (Hippomorphia), family (Equidae), subfamily (Equinae), and genus (Equus) and differ only in their species. Graphics created with BioRender.com.

Figure 4 displays the relationship between present and potential future mammalian milk sources for infant formula production discussed in this part of the review. Cow, goat, sheep, and camel belong to the order Artiodactyla; cow, goat, and sheep belong to the Ruminantia suborder and Bovidae family, while camel belongs to Tylopoda suborder and Camelidae family [259,260]. In addition, cow belongs to Bovinae subfamily, while goat and sheep belong to Caprinae subfamily. Further, goat belongs to Capra genius, and sheep belongs to Ovis genus [261]. Donkey and horse belong to another order called Perissodactyla and further belong to the same suborder (Hippomorphia), family (Equidae), subfamily (Equinae), and genus (Equus) and differ only in their species [262]. Artiodactyla and Perissodactyla orders are equally distanced from the Primates order (human).

Cross-reactivity between cow’s milk proteins and counterpart proteins from other mammalian milk is an important factor when evaluating the usability of non-cattle milk in CMA prevention and management. Therefore, in Table 2, the amino acid sequence identity of allergens from cow’s milk and their counterpart proteins in goat, sheep, camel, donkey, horse, and human milk is presented. Overall, from Table 2, it can be seen that goat and sheep milk proteins have a higher sequence identity with cow’s milk allergens than proteins from camel, donkey, horse, and human milk. Furthermore, donkey and horse proteins have in general a lower sequence identity with cow’s milk allergens than proteins from camel milk.

For CMA prevention, a certain degree of cross-reactivity between cow’s milk and other non-cattle milk proteins is needed. Hence, in all probability, goat and sheep milk proteins would be a better choice for CMA prevention than camel, donkey, and horse milk proteins due to the high amino acid sequence identity with cow’s milk proteins (Table 2). However, no studies have yet analysed the usability of non-cattle milk on CMA prevention. Thus, in the following sections, we will discuss the suitability of milk proteins from goat, sheep, camel, donkey, and horse milk as a protein source in alternative infant formulas for CMA management.

### 6.1. Goat Milk

Milk from *Capra hircus* (goat) is widely available and used especially in the Mediterranean area of Europe as well as in some Western Europe countries, Asia, Australia, and New Zealand. Goat belongs to the Bovidae family, along with cow and sheep, and together with sheep, it belongs to the Caprinae subfamily (Figure 4). Goat milk is used as raw or pasteurised regular milk, in cheese, and in yoghurt production [264] as well as in the production of infant formulas as a source of proteins and micro and macro nutrients [265]. It contains a comparable amount of total protein to cow’s milk, with a slightly higher ratio of caseins to whey proteins, being 84:16 compared to 80:20 for cow’s milk [119]. Moreover, the profile of individual proteins differs, where it has been shown that goat milk contains significantly lower amount of α_s1_-casein but significantly higher amount of α_s2_-, β-, and ĸ-casein compared to cow’s milk [265,266]. On the other hand, the amount of specific whey proteins was found to be comparable for cow’s and goat’s milk [265]. Cow’s and goat’s milk proteins possess high amino acid sequence identities, as shown in Table 2. The amino acid sequence identities range between 85 and 95%, being slightly higher for whey proteins compared to caseins.

In 2012, European Food Safety Authority (EFSA) concluded that goat milk is a suitable source of proteins for infant formula [257]. Initially, goat milk was suggested as an alternative to hypoallergenic infant formulas for cow’s milk allergic patients [267,268,269], but in recent years, there has been growing evidence supporting that infant formula based on intact goat milk proteins is not suitable as an alternative to hypoallergenic infant formulas for the management of CMA. In fact, DRACMA guideline [106] as well as an opinion by EFSA Scientific Panel [257] highlighted the importance of avoiding goat milk for CMA management.

Several studies have shown that IgE-mediated cow’s milk allergic patients manifest cross-reactivity towards goat milk proteins. Based on in vivo and ex vivo analyses, they concluded that only few patients with CMA can tolerate goat milk and that most react to goat milk [270,271,272]. Conversely, there are some single cases reporting a tolerance to cow’s milk in patients allergic to goat milk [273,274,275,276], indicating development of IgE specific for epitopes only present in goat milk proteins but absent in cow’s milk proteins. For example, a study by Bernard et al. found an absence of cross-reactivity in patients allergic to goat milk with tolerance to cow’s milk using β-casein [277]. The study concluded that the specificity of the IgE response to goat milk β-casein with concomitant lack of response to cow’s milk β-casein was a result of difference in only three amino acids in the domain between amino acid 49 and 79, indicating that even small differences may indeed have a great impact on the IgE-binding capacity [277]. At present, no goat milk proteins are registered as allergens in the AllergenNomenclature [56] although studies have been reporting cases of goat milk allergy [273,274,275,276]. Allergenicity of goat milk has also been evaluated in animal models, where goat milk was shown to inhere a lower allergenicity than cow’s milk [278,279].

Based on the current evidence, goat milk infant formula should be avoided in cow’s milk allergic patients and should not be recommended for CMA management.

### 6.2. Sheep Milk

Milk from *Ovis aries* (sheep) is mainly available in countries such as China, New Zealand, Turkey, Greece, Syria, and Romania [280,281]. Together with cow and goat, sheep belongs to the Bovidae family, and together with goat, it belongs to the Caprinae subfamily (Figure 4). Sheep milk contains a higher amount of total protein compared to cow’s milk, with a ratio of caseins to whey proteins comparable to that of cow’s milk, i.e., 80:20 [281]. It contains a different profile of the specific proteins, with a higher amount of β- and α_s2_-casein and lower amount of ĸ- and α_s1_-casein than cow’s milk [281]. Similar to goat milk, sheep milk contains high amino acid sequence identities with counterpart cow’s milk proteins, ranging between 85 and 95% and being slightly higher for whey proteins than caseins (Table 2).

Currently, sheep milk-based infant formulas are not approved in EU and hence are not commercially available but are available in China and New Zealand [282,283]. From the perspective of CMA management, currently, there is lack of studies evaluation usability of sheep milk. Yet, based on the large degree of homology between sheep and cow’s milk proteins, similar to the homology between goat and cow’s milk proteins, it must be anticipated that most cow’s milk allergic infants may react to sheep milk-based infant formulas. However, several cases have been reported with allergic reactions toward sheep milk proteins after sheep cheese consumption in individuals who could tolerate and had no allergic reactions toward cow’s milk [284,285,286,287,288]. Currently, no sheep milk proteins are registered as allergens in the AllergenNomenclature [56]

### 6.3. Camel Milk

Milk from *Camelus dromedaries* (camel) is an important source of nutrition in arid and semi-arid regions because camels can produce much more milk while on poor feed and lack of water than any other species [289,290]. In these regions, camel milk is used as raw or pasteurised regular milk or is used in dairy product manufacture for yoghurt, soft cheese, or ice creams. Together with cow, goat, and sheep, camel belongs to the Artiodactyla order but to a different family, namely the Camelidae family (Figure 4) [290]. In general, camel milk contains comparable amount of total proteins to cow’s milk [291]. However, the ratio between caseins and whey proteins is different from that in cow’s milk, with 74:16 in contrast to the 80:20 for cow’s milk [129]. In addition, camel and cow’s milk differ in their specific protein profile. First of all, β-lactoglobulin (Bos d 5), a protein found in the cow’s milk whey fraction, also known as one of the major allergens [292], is not present in camel milk [291,293,294]. Moreover, camel milk contains lower amount of α_s1_- and ĸ-casein and higher amount of β-casein compared to cow’s milk. In addition, the amount of α-lactalbumin and serum albumin is higher in the whey fraction of camel milk compared to the whey fraction of cow’s milk [291]. The amino acid sequence identities between camel and cow’s milk proteins range between 47 and 81%, being higher for whey proteins than caseins (Table 2).

Camel milk has gained an increasing interest in the last decade as a potential suitability source for infant formula manufacture, including manufacture of infant formulas for CMA management [290,295,296]. This is mainly due to its different profile of proteins and relatively low amino acid sequence identities with cow’s milk proteins especially in comparison to goat and sheep milk proteins [263]. At present, no infant formula based on camel milk is available on the EU market; however, in the Middle East, a stage three toddler formula based on camel milk is commercially available.

At present, there is a number of clinical trials evaluating the usefulness of camel milk as an alternative milk for patients allergic to cow’s milk, and the results are consistent. A study by Navarrete-Rodríguez et al. showed no clinical symptom manifestation after two weeks of consumption of camel milk in patients with confirmed CMA [297]. Moreover, several studies using in vivo method, such as SPT, showed a low level of reactivity towards camel milk, with <20% of the cow’s milk allergic patients reacting [298,299,300].

In addition, there are a number of studies performing ex vivo analyses using blood from cow’s milk allergic patients for antibody reactivity evaluation, concluding reduced or no reactivity of specific IgE towards camel milk proteins [301,302,303,304].

Currently, there is one case that reported anaphylaxis after camel milk consumption in an atopic child who had never experienced allergy to cow’s milk proteins [305]. At present, no proteins from camel milk are registered as allergens in the AllergenNomenclature [56].

An animal study evaluating cross-reactivity between cow’s and camel milk proteins showed that there was low cross-reactivity between camel and cow’s milk proteins, with lower cross-reactivity between caseins than between whey proteins [263]. The study also showed that the linear epitopes were predominant in casein cross-reactivity, while conformational epitopes prevailed in whey protein cross-reactivity.

Camel milk may have a potential to be used as a source of proteins in infant formulas for CMA management. However, further investigations are required.

### 6.4. Donkey Milk

Milk from *Equus asinus* (donkey) is mostly common in the Mediterranean countries, such as Spain, Greece, France and Italy, as well as Asian and African countries [306]. Together with the horse, donkey belongs to another order than cow, goat, sheep, and camel, namely the Perissodactyla order (Figure 4).

Donkey milk contains around two times less proteins in comparison to cow’s milk [307]. In addition, it has a very different casein-to-whey protein ratio, with 58:42 in contrast to 80:20 for cow’s milk [119,308]. Donkey milk contains a lower amount of α_s2_-casein but significantly higher amount of α_s1_-, ĸ-, and β-casein than cow’s milk [308]. In general, the protein sequence identities between donkey and cow’s milk proteins are low when compared with proteins from goat or sheep milk. The amino acid sequence identities range between 47 and 74% for whey proteins and between 46 and 60% for caseins (Table 2).

As a non-cattle milk, donkey milk is gaining increasing interest, especially in Italy [307], for its potential usability in infants with CMA and thus for the future application as a protein source in infant formula manufacture. Several clinical studies have been performed to evaluate the safety of donkey milk in cow’s milk allergic patients using in vivo or ex vivo methods. The outcome of the studies were consistent, where tolerance to donkey milk after OFC was reported in more than 80% of the cow’s milk allergic patients enrolled in all studies [309,310,311,312,313].

Vita et al. compared the level of tolerance towards goat and donkey milk in patients with atopic dermatitis and CMA [314] and showed that donkey milk was tolerated by 88% of patients in comparison to none for goat milk and that consumption of donkey milk improved the atopic dermatitis.

At present, there are a number of cases reported in relation to donkey milk protein allergy without concomitant CMA, including two patients who developed symptoms after donkey milk consumption [315,316] and one manifesting clinical symptoms after inhalation, showing respiratory allergy [317]. In addition, a case of skin contact allergy was reported where a patient developed urticaria after using donkey milk containing cosmetics [315]. Based on two cases reported by Martini et al. [308,315], lysozyme was identified as an allergen in donkey milk and included in the AllergenNomenclature [56].

Based on the current evidence showing a high level of tolerance to donkey milk in patients with CMA, donkey milk may be a potential source of proteins in future infant formulas for CMA management. However, further investigations are needed.

### 6.5. Horse Milk

Milk from *Equus ferus caballus* (horse) is mainly popular in countries such as Mongolia, Kazakstan, Kyrgyzstan, and Tajikistan [318,319]. Together with donkey, horse belongs to another order than cow, goat, sheep, and camel, namely the Perissodactyla order (Figure 4).

Horse milk contains two times less protein than cow’s milk and has a comparable casein-to-whey protein ratio to that of donkey milk, i.e., 56:44, which is very different from that of cow’s milk at 80:20 [119]. Horse milk has a lower α_s1_- and α_s2_-casein content and a higher α-lactalbumin content than cow’s milk [318]. The amino acid sequence identities with cow’s milk proteins range between 51 and 74% for whey proteins and between 46 and 57% for caseins (Table 2). Like donkey milk, horse milk is gaining increased interest for its potential usability for cow’s milk allergic infants and children.

A study by Businco et al. showed that horse milk was tolerated by 96% children with CMA by means of an OFC [320], and in a study by Fotschki et al. using an animal model, it was shown that horse milk consumption decreased total IgE level in mice sensitised to cow’s milk [321]. In another animal model, the allergenicity of horse milk was shown to be lower than the allergenicity of cow’s and goat milk [322].

Cases of horse milk allergy without concomitant CMA have been reported. One case report described skin contact allergy after application of a body cream containing horse milk as an ingredient with manifestation of swelling and itchiness but also horse milk α-lactalbumin-positive IgE in serum [323]. Moreover, two cases of horse milk allergy have been reported after its consumption, without concomitant CMA [324,325].

Horse milk lysozyme is registered as an allergen in the AllergenNomenclature [56] due to a 99% sequence identity with donkey milk lysozyme [315]. In addition, horse serum albumin has been registered as an airway allergen [56,326]. Yet, as serum albumin is a protein also found in milk, patients with confirmed horse serum albumin inhalation allergy should also avoid horse milk consumption.

Current evidence indicates that horse milk, just as donkey milk, possesses a high level of tolerance in patients with CMA. However, more studies are required for a further evaluation of its usefulness as a protein source in infant formulas for CMA management.

## 7. Plant-Based Infant Formulas

Infant formulas, as substitutes to breastmilk, are largely based on dairy proteins. Yet, in recent years, there has been a great focus on alternative protein sources of plant origin—not only as a substitute to cow’s milk-based formulas for infants suffering from CMA or cow’s milk intolerance but also for taste preference, vegan habits, environmental, climate, and ethical reasons [9,10]. Indeed, there is an immense focus on providing more sustainable and climate-friendly dietary solutions for the future [327,328,329].

In general, the demand for plant-based beverages has increased throughout the world in the last years [330,331,332] and can be divided into five categories: cereal-based (oat, rice, corn, spelt), legumes-based (soy, peanut, lupin, cowpea, chickpea), nut-based (almond, coconut, cashew, hazelnut, Brazil nut, pistachio), seed-based (sunflower, sesame, hemp), and pseudocereal-based (quinoa, teff, amaranth) beverages [10,144,333,334,335]. It has been reported that parents and caretakers are increasingly feeding infants and young children with such plant-based beverages as alternatives to cow’s milk-based products, including as substitutes for cow’s milk-based infant formulas [336,337]. The quality of plant-based alternatives varies and may not necessarily address the nutritional requirements of infants and small children [144,331,333,338]. Thus, it appears that there is no health benefit of plant-based alternatives to cow’s milk-based products in small children but rather a potential health risk related to frequent consumption of these plant-based alternatives if the child’s diet is not properly managed [331,338]. In fact, case-based evidence with severe malnutrition caused by plant-based beverage feeding in infants down to 1 months of age has been reported [337,338] where, in some cases, the infants were fed with the plant-based beverages already from birth [338].

**Figure 5:**
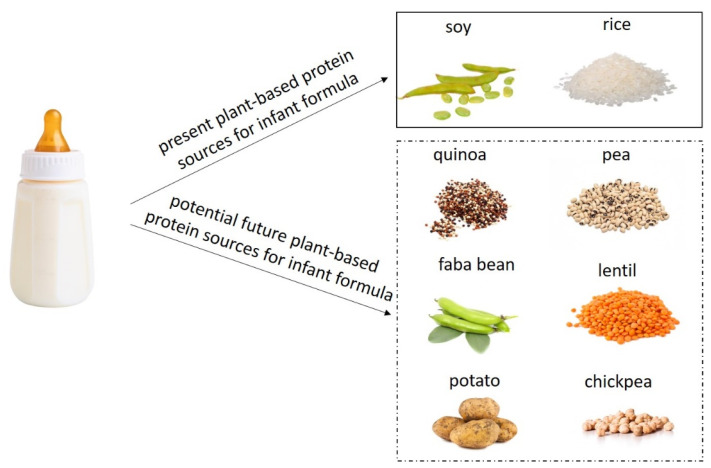
Present and potential future plant-based protein sources for infant formula manufacture. Present plant-based protein sources for commercially available infant formula manufacture are soy and rice. Potential future plant-based protein sources suggested for infant formula manufacture are quinoa, pea, faba bean, lentil, potato, and chickpea. Pictures were purchased from Colourbox.com.

Indeed, there has been recommendation against plant-based beverages for small children [338], and for infants up to an age of 12 months, it is recommended only to use appropriate commercial infant formulas as alternatives to breastmilk [336]. Only a few commercially available infant formulas based on plant proteins exist, and these are manufactured from either soy or rice proteins (Figure 5). In the EU, the only source of protein allowed in infant and follow-up formulas are cow’s milk, goat milk, soy, as well as hydrolysed proteins [7].

For infants with severe CMA that cannot tolerate eHF, alternatives to AAF are soy- and hydrolysed rice-based infant formulas. These infant formulas are, in general, well tolerated and considered a second choice for cow’s milk allergic infants and small children in some countries [1]. Yet, ESPGHAN and EAACI recommend against the use of soy protein-based formulas in infants below the age of 6 months [23,104,339]. Similar to eHF, plant-based infant formulas for management of CMA in infants should also be tolerated by at least 90% of the children with CMA, with a confidence interval of 95% [6,80]. However, in some cases, plant-protein based formulas may not prove hypoallergenic for cow’s milk allergic infants [336].

### 7.1. Soy-Based Infant Formulas

Soybean is a legume crop originating from East Asia with a high-quality content of proteins comprising up to 40% of the dry weight [339,340]. Soy-based infant formulas are available in many countries throughout the world, with the largest market being in North America [341]. Soy-based infant formulas have been commercially available for more than a century although they have changed throughout this time [340,342,343]. At first, the soy-based infant formulas were altered from being based on soy flour to soy protein isolate in order to obtain a higher digestibility and a lower content of fibres and phytates [343,344]. Later, the soy-based infant formulas were fortified with the amino acids methionine, taurine, and carnitine as well as with choline and inositol [342,343]. Most recently, the soy-based infant formulas have been supplemented with LCPUFAs [344]. Despite the initiatives to improve soy-based infant formulas over time in order for them to be safe and to meet the nutritional need of infants comparable to that of cow’s-milk-based infant formulas [118,342,343], concerns have been raised regarding potential risks due to the phytate and phytoestrogen content as well as nutritional deficiencies [1,339,342]. Yet, based on a meta-analysis, Vandenplas et al. concluded that soy-based infant formulas are a safe alternative to cow’s milk-based infant formulas [343].

Soy allergy is less prevalent than CMA although it affects around 0.3–0.4% of small children [345,346], and according to AllergenNomenclature, eight allergens have been identified [56]. However, as soy-based infant formulas do not contain cow’s milk proteins and lactose, it may be a choice for infants suffering from CMA or cow’s milk intolerance, and for most cow’s milk allergic infants, soy-based infant formulas are also well-tolerated [144]. Before the introduction of eHF on the market, it was the only formula available for the feeding of infants with CMA [339,347]. However, co-sensitisation to cow’s milk and soy proteins is common, whereas cross-reactivity between cow’s milk and soy proteins is not [346] even though it has been demonstrated [348]. It has been reported that up to 50% of cow’s milk allergic infants may react to the soy-based infant formulas [339,347] although different studies based on OFC have shown lower yet varying results, revealing clinically relevant reactions to soy or soy-based infant formula in 3% [349], 7% [350], 10% [351], or 14% [352] of cow’s milk allergic infants, respectively.

The use of soy-based infant formulas in the prevention of atopic diseases in high-risk infants seems controversial [339,347], with studies showing some prophylactic effect of soy-based infant formulas when compared to cow’s milk-based formulas [353,354], whereas other studies did not show such effect [355,356]. Yet, in a meta-analysis, Osborn and Sinn concluded that soy-based infant formulas cannot be recommended for use in the prevention of food allergies in high-risk infants [357], which is generally supported by most guidelines [358]. For example, EAACI recommends against using soy-based formula in the first 6 months of life as a means of preventing food allergy [81]. Controversies also exist for the acquisition of tolerance to cow’s milk when soy-based infant formulas are used in the management of CMA, where one study reported that soy-based infant formula was more effective than eHF in tolerance acquisition [359], whereas another study showed that an eHF was more effective than was a soy-based infant formula [360], and a third study showed no differences between the formula choice on tolerance acquisition [355].

Due to the perceptual nutritional disadvantages and allergenic potential of soy-based infant formulas, ESPHGAN, EAACI, and AAP do not recommend giving soy-based infant formulas to infants below the age of 6 months [23,104,118,339]. Yet, ESPGHAN and AAP state that soy-based infant formulas may be considered in infants above the age of 6 months when complementary feeding has been initiated and in the absence of soy allergy for infants suffering from CMA and when parents wish to exclude products of animal origin or believe that eHFs are too expensive [104,339].

### 7.2. Hydrolysed Rice-Based Infant Formulas

Rice is a cereal believed to originate from Asia and has a rather low content of proteins comprising around 8% of the dry weight [361,362,363]. Rice-based infant formulas have for two decades been available in Spain, Italy, and France, where they are categorised as “foods for special medical purposes” (FSMP) [144,364,365] according to European law [366,367]. These are foods intended for dietary management, under medical supervision, of patients who suffer from certain diseases, disorder, or medical condition [366]. A requirement for formulas based on rice is that these formulas shall be based on hydrolysed rice proteins to obtain higher water solubility and digestibility as well as be fortified with the amino acids lysine, threonine, and tryptophan [365]. While hydrolysed rice-based infant formulas are available in Spain, Italy, and France, they are still not available in other European countries as well as in the U.S., Canada, Australia, and New Zealand but are, on the other hand, emerging in a growing number of African, Asian, and South American countries [364].

Commercially available hydrolysed rice-based infant formulas are in general well-tolerated and support the normal growth of infants [365,368,369] and have been reported to be growing in popularity due to their proven safety and due to being a cheaper choice than eHF [144,364,365].

The prevalence of rice allergy in small children is common in countries where it is frequently eaten but is generally low in Western countries [144,365,370,371], and according to AllergenNomenclature, only two rice allergens have been identified being categorised as respiratory allergens [56]. This as well as the absence of cross-reactivity between rice and cow’s milk proteins makes rice-based formulas well tolerated in children with CMA, and only a limited number of cases of allergic responses toward hydrolysed rice-based infant formulas has been implied [364]. In two studies, it has been reported that cow’s milk allergic infants showed reactivity to the hydrolysed rice-based infant formulas with specific IgE > 0.35 kU/L or with positive SPT although no clinical reactivity was observed upon OFC [370,372], whereas in another study, no reactivity to the hydrolysed rice-based infant formula was revealed [373].

When it comes to acquisition of tolerance to cow’s milk when hydrolysed rice-based infant formulas are used in the management of CMA, there are conflicting evidence, where one study showed no differences when compared to eHF [373], another study showed that hydrolysed rice-based infant formulas were more effective than eHF [359], and a third study showed the eHF to be more effective than hydrolysed rice-based infant formula in acquisition of tolerance to cow’s milk [360].

Hydrolysed rice-based infant formulas have, in general, proven to be a safe choice for cow’s milk allergic infants, and the DRACMA guidelines suggest that hydrolysed rice-based infant formulas may be an equivalent to AAF as second choice for infant formula feeding if eHFs cannot be tolerated for the management of CMA [296]. Yet, increasing amounts of data are being published even supporting the use of hydrolysed rice-based formulas as a possible first choice for infants with CMA [107]. However, ESPGHAN, EAACI, and AAP do not mention hydrolysed rice-based infant formulas in their guidelines [23,80,104].

### 7.3. Potential Future Plant-Based Infant Formulas

In contrast to soy-based and hydrolysed rice-based infant formulas, where there is a great amount of literature available, there is not much literature on other plant-based alternatives to cow’s milk based infant formulas. Soy- and hydrolysed rice-based formulas are presently the only infant formulas nutritionally adapted for infants that are commercially available [107]. Yet, in the last decade, there has been an increasing interest in investigating new and potential future plant-based infant formulas either as complete plant-based infant formulas or as partial plant-based infant formulas, where only a proportion of the cow’s milk proteins are substituted with plant proteins.

Several plants have been suggested as potential suitable protein sources for new infant formulas, these being quinoa [374], pea [375,376,377], faba bean [375,376,377], lentil [378], potato [376,379], and chickpea [380,381], as shown on Figure 5. Nevertheless, before any of these plant-based protein sources can be used in infant formulas, they would need to comply with the Regulation EU 2016/127 [114], and for some, they may even be regarded as novel foods, as new processing procedures may be a necessity to provide protein isolates and hence require an EU authorisation as a novel food [382].

Most of the studies concerning new plant-based infant formulas have focused primarily on physicochemical and functional properties as well as digestibility, as integration of new protein sources in formulas for infant nutrition rely on some “standard” properties, such as solubility, emulsification, and stability, as well as nutritional quality required to meet the needs of infants [114,383].

For legumes, lentil proteins have been suggested as an alternative to cow’s milk proteins for infant formulas due to a high protein content of around 20–30% and a good amino acid profile [378]. In a recent study, Alonso-Miravalles et al. [378] investigated the physicochemical properties of a lentil protein-based formulation in comparison to two conventional plant-based infant formulas: one based on soy protein and one based on rice proteins. They concluded that from a physicochemical and nutritional perspective, lentil proteins are a good alternative to other sources of plant proteins for infant formulas [378]. However, allergenicity to lentil seems to be well documented in some countries and may lead to severe allergic reactions [384,385], and according to the AllergenNomenclature, three lentil allergens have been identified [56]. Lentil allergy has been reported as one of the most common non-priority (emerging) food allergies, which could be envisioned to increase in prevalence due to its increasing popularity [386].

The legume faba bean is also known as fava bean or broad bean. In studies of partial substitution of cow’s milk proteins with faba bean proteins in infant formulas [375,376,377], it was reported that physicochemical properties of the formula were affected to some degree by the protein substitution [377]. It was shown that the digestibility of the formula was higher by substituting 50% of the cow’s milk proteins with faba bean protein in a dynamic in vitro model [375], whereas there were no significant differences observed in a static in vitro digestion model [376]. Overall, partial substitution of cow’s milk proteins with faba bean proteins resulted in formula physicochemical and digestibility properties more closely resembling those of fully cow’s milk-based infant formula than if cow’s milk proteins were substituted with rice proteins [376]. Thus, Le Roux et al. concluded that faba bean proteins could be a good candidate for partial substitution of cow’s milk proteins in infant formulas [375,376] although process parameters would need to be optimised to meet infant formula quality criteria [377]. Allergy to faba beans has only been demonstrated in few studies; yet, these indicate that faba beans contain clinically relevant allergens [387,388] although no faba bean allergens are listed in the AllergenNomenclature [56].

Similarly, the legume pea has also been studied in a partial substitution of cow’s milk proteins for infant formulas [375,376,377], with the main difference compared to faba beans being that substituting 50% of cow’s milk proteins with pea proteins resulted in a lower digestibility [375]. Pea allergy is well documented [389], and according to the AllergenNomenclature, pea contains three identified allergens [56]. Like lentil allergy, pea allergy has been reported as one of the most common non-priority (emerging) food allergies [384,386], and with the increasing use of pea protein isolate in various foods, it must be anticipated that allergy to pea will increase [384,389].

Chickpea, which is also a legume, has been suggested as a potential future protein source in infant formulas [380,381]. In evaluating the nutritional value of chickpea-based formulations, it was concluded that chickpea-based formulas may be a potential future alternative to cow’s milk-based formulas for infants above the age of 6 months [380,381]. Allergy to chickpea has been reported in few studies, with identification of two allergens [384,385], though only one chickpea allergen is listed in the AllergenNomenclature [56]. For pseudocereals, quinoa has been suggested as an alternative source to cow’s milk proteins in follow-up formulas due to its high-quality protein content [374]. Quinoa seems to be increasingly appreciated as an excellent gluten-free protein source for a wide range of consumers, including infants [374,390,391]. Yet, concerns have been raised regarding the high amount of saponins in quinoa, which inhere adjuvant capacity and may affect intestinal permeability [392]. Only few studies have investigated allergy to quinoa proteins, showing that quinoa may contain allergenic proteins [393,394,395,396]. However, no quinoa allergens are listed in the AllergenNomenclature [56]. Nevertheless, it should be acknowledged that since quinoa has not been a standard part of the Western diet, larger amounts and frequent consumption of quinoa might enhance the development of allergies.

Potato is a root vegetable commonly ingested throughout the world. Although potato allergy is uncommon, cases of potato allergy have been reported [397], and according to the AllergenNomenclature, four potato allergens have been identified [56]. Recently, a patent application on an infant formula for cow’s milk allergic infants wherein the major source of protein is potato proteins has been filed [WO2018050705] [379].

Currently, plant-based infant formulas based on other proteins than those derived from soy and hydrolysed rice are not allowed in infant formulas according to the Regulation EU No 609/2013 [7]; however, in the latest Regulation EU 2016/127, it is stated that in order to ensure innovation and product development, other ingredients not covered by the specific requirement of the Regulation should be possible, provided their suitability for infant feeding has been demonstrated and authorised [114]. Recently, some toddler formulations have been marketed based on pea, rice, buckwheat, and almond that meet the nutritional need of toddlers [107].

## 8. Conclusions

In this review, we provided an overview of current and potential future options for protein sources in infant formulas in the context of CMA prevention and management. Breastfeeding should always be a first choice of infants feeding both in general as well as in CMA prevention and management. Cow’s milk-based infant formulas are the main substitute to mother’s milk if breastfeeding is not possible, insufficient, or not chosen. In addition to cow’s milk-based infant formulas, infant formulas based on goat milk, soy, or hydrolysed rice proteins are also available on the EU market.

There are presently no specific recommendations for use of any particular infant formula for CMA prevention, but it was, until recently, recommended to use pHF for the prevention of CMA in high-risk infants. For CMA management, eHFs are currently recommended as a first choice; however, if the eHF is not tolerated or if the infant suffers from severe CMA, AAF or alternatively a hydrolysed rice-based formula may be a second choice. Yet, infant formulas based on modified cow’s milk proteins, other mammalian milk proteins, and plant-based proteins have been investigated as potential future protein sources for infant formulas both in general and for cow’s milk allergic infants.

Processing technologies in addition to hydrolysis, such as heat treatment, fermentation, HP, and irradiation, are methods known to modify proteins by means of breakdown, denaturation, aggregation, oxidation, and glycation and thus have the potential to reduce allergenicity.

Whereas goat- and sheep-milk-based infant formulas may be good alternatives to conventional cow’s milk-based infant formulas, they are not a suitable choice for CMA management due to the high homology of their proteins to cow’s milk allergens and hence the great risk of cross-reactivity. Camel, donkey, and horse milk may, however, provide a better alternative for CMA management due to the lower protein homology and hence lower cross-reactivity to counterpart cow’s milk allergens. Camel milk, which lacks the protein β-lactoglobulin, would have the potential to be used for infants allergic to primarily cow’s milk β-lactoglobulin. Yet, the amount of evidence for the suitability of alternatively mammalian milk for use in cow’s milk allergic infants is still limited, and thus, more research is needed.

Infant formulas based on soy and hydrolysed rice proteins are plant based-infant formulas presently available on the market in some countries and recommended as a potential second choice for CMA management in some countries. However, ESPGHAN, EAACI, and AAP do not recommend the use of soy-based infant formulas in infants below 6 months of age. Infant formulas based on alternative plant proteins have, in the last decade, gained an increasing interest as a sustainability and vegan alternative to milk-based infant formulas, and several plants have been suggested as a protein source for future infant formulas. With the current focus on more sustainable and climate-friendly dietary solutions, we could foresee that much more research would be conducted for evaluation of the suitability of plant-based infant formulas not only for the CMA management but as a general choice for infant nutrition.

## Figures and Tables

**Table 1:** Cow’s milk allergens and their characteristics. Table modified from [53].

Cow’s Milk Fraction	Protein	Allergen Name	Content (%)	Size (kDa)	Major/Minor Allergen	S-S Bridges
Casein (80%)	α_s1_-casein	Bos d 9	32	23.6	Major	0
	α_s2_-casein	Bos d 10	10	25.2	Major	0
	β-casein	Bos d 11	28	24	Major	0
	ĸ-casein	Bos d 12	10	19	Major	1
Whey (20%)	α-lactalbumin	Bos d 4	5	14.2	Major	4
	β-lactoglobulin	Bos d 5	10	18.3	Major	2 + 1 free
	Serum albumin	Bos d 6	1	66.3	Minor	17 + 1 free
	Immunoglobulins	Bos d 7	3	160	Minor	number varies ^1^
	Lactoferrin		<1	80	Minor	16

^1^ The number of disulphide (S-S) bridges in immunoglobulins varies depending on their classes as well as subclasses [54].

**Table 2:** Amino acid sequence identity percentage (%) between cow and goat, sheep, camel, donkey, horse, and human milk proteins. Table modified from [263].

Protein	Goat	Sheep	Camel	Donkey ^1^	Horse ^1^	Human
α_s1_-casein	91	91	67	57	55	33
α_s2_-casein	88	88	47	46	46	NA
β-casein	88	89	56	60	57	55
ĸ-casein	85	85	58	57	56	52
α-lactalbumin	95	95	60	47	60	74
β-lactoglobulin	93	93	NA	52	51	NA
Serum albumin	88	91	81	74	74	76
Lactoferrin	92	92	75	73	73	70

^1^ Donkey and horse milk proteins were not included in amino acid sequence identity % table from [263]. Therefore, sequence alignments between cow’s milk proteins and donkey and horse milk proteins were performed using CLS Main Workbench 8.0 and Uniprot and NCBI database. NA, not available. Accession number: β-casein: Cow: AAA30431; Donkey: XP_044622644; Horse: NP_001075321. α_S1_-casein: Cow: AAA30429; Donkey: XP_014708642; Horse: AAK83668. α_S2_-casein: Cow: NP_776953; Donkey: XP_044622647; Horse: NP_001164238. ĸ-casein: Cow: CAA33034; Donkey: XP_014702750; Horse: AAK83669. α-lactalbumin: Cow: CAA29664; Donkey: XP_014705618; Horse: P08896. β-lactoglobulin: Cow: CAA32835; Donkey: P13613; Horse: AAC95385. Serum albumin: Cow: CAA41735; Donkey: AAV28861; Horse: P358747. Lactoferrin: Cow: AAA30610; Donkey: XP_044610851; Horse: NP_001157446.

## Data Availability

Not applicable.
